# Risk Factors and a Prediction Model of Lateral Lymph Node Metastasis in CN0 Papillary Thyroid Carcinoma Patients With 1–2 Central Lymph Node Metastases

**DOI:** 10.3389/fendo.2021.716728

**Published:** 2021-10-15

**Authors:** Yuanyuan Wang, Chang Deng, Xiujie Shu, Ping Yu, Huaqiang Wang, Xinliang Su, Jinxiang Tan

**Affiliations:** ^1^ Department of Endocrine and Breast Surgery, The First Affiliated Hospital of Chongqing Medical University, Chongqing, China; ^2^ Department of Hepatobiliary, Breast and Thyroid Surgery, The People’s Hospital of Nanchuan, Chongqing, China

**Keywords:** cN0, PTC, LLNM, CLNM, LASSO

## Abstract

**Background:**

Papillary thyroid cancer (PTC) in clinically lymph node-negative (cN0) patients is prone toward lymph node metastasis. As a risk factor for tumor persistence and local recurrence, lateral lymph node metastasis (LLNM) is related to the number of central lymph node metastases (CLNMs).

**Methods:**

We performed LLNM risk stratification based on the number of CLNMs for cN0 PTC patients who underwent thyroidectomy and lymph node dissection between January 2013 and December 2018. A retrospective analysis was applied to the 274 collected patients with 1-2 CLNMs. We examined the clinicopathological characteristics of the patients and constructed a LASSO model.

**Results:**

In the 1–2 CLNM group, tumors >10 mm located in the upper region and nodular goiters were independent risk factors for LLNM. Specifically, tumors >20 mm and located in the upper region contributed to metastasis risk at level II. Hashimoto’s thyroiditis reduced this risk (*p* = 0.045, OR = 0.280). Age ≤ 30 years and calcification (microcalcification within thyroid nodules) correlated with LLNM. The LASSO model divided the population into low- (25.74%) and high-risk (57.25%) groups for LLNM, with an AUC of 0.715.

**Conclusions:**

For patients with 1–2 CLNMs, young age, calcification, nodular goiter, tumor >10 mm, and tumor in the upper region should alert clinicians to considering a higher occult LLNM burden. Close follow-up and therapy adjustment may be warranted for high-risk patients.

## Introduction

Thyroid cancer (TC) is a common endocrine malignancy with an incidence that has constantly increased for decades (1). In 2018, the growth rate rose to 3.1%, with 567,233 new cases (2) and the population experiencing morbidity displaying a younger trend (3). As a major pathological type of TC, papillary thyroid carcinoma (PTC) has a favorable prognosis (4). However, lymph node metastasis (LNM) occurs even in the early stage, with reported incidence rates ranging from 40% to 90% (5). Moreover, LNM correlates with tumor persistence and recurrence and even a poor prognosis (6). It is also reported to occur in more than 80% of recurrence cases (7).

At present, the indications and dissection scopes of lymph node dissection (LND) are still controversial, especially for clinically lymph node-negative (cN0) PTC. In fact, the accuracy of preoperative assessment for cN0 PTC patients is 67.6% and the sensitivity is low (8). Studies show that the incidence of occult central lymph node metastasis (CLNM) ranges from 30% to 80%, with that of occult lateral lymph node metastasis (LLNM) ranging from 18.6% to 64% (8–10). Of note, Asians have a much higher incidence than other populations. According to National Comprehensive Cancer Network (NCCN) (11) and American Thyroid Association (ATA) guidelines (12), experts disagree on prophylactic central lymph node dissection (pCLND), although the Japanese Association of Endocrine Surgeons and the Japanese Society of Thyroid Surgeons recommend routine pCLND (13). By consensus, prophylactic lateral lymph node dissection (pLLND) is not recommended.

China accounts for 37.72% of the world’s new cases (14). Over 80% of Chinese patients have LNM and the 5-year relative survival rate is 84.3%, which is much lower than that in the United States according to the Database Technology Conference China (DTCC) report. Based on research, Chinese expert consensus advises pCLND and selective LLND for high-risk patients with CLNM (15). Many reports have revealed that the number of CLNMs is an independent risk factor for LLNM. The cutoff value differs across studies, ranging from 2 to 3. In general, cN0 PTC patients have a low risk of skip metastasis (central lymph node negative and lateral lymph node positive) (16) and a high risk of LLNM (60%–75%) with ≥3 pathologic CCLMs (17, 18). However, there are few reports on the risk factors for LLNM in CN0 PTC patients with 1–2 pathologic CCLMs.

In this retrospective study, cN0 PTC patients with 1–2 pathologic CCLMs had a median LLNM incidence compared to the skip metastasis and ≥3 CCLM groups. We also investigated the risk factors for LLNM in cN0 PTC patients with 1–2 CCLMs and constructed a prediction model using the LASSO method.

## Materials and Methods

### Patients

This retrospective study enrolled 1,033 patients at the Department of Endocrine and Breast Surgery from January 2013 to 2018 and was approved by Medical Ethics Committee, the First Affiliated Hospital of Chongqing Medical University. Patients were diagnosed by fine-needle aspiration biopsy (FNAB) and intraoperative frozen and postoperative pathology examined pathologically by three pathologists. Before surgery, physical examination, laryngoscopy, and neck ultrasound were conducted by two experienced ultrasound doctors. The inclusion criteria were as follows: cN0 PTC, complete clinicopathologic data, and primary surgery with ipsilateral lobectomy, CLND, and LLND, including levels II, III, and IV. Intraoperative frozen pathology of CLNs was performed to determine metastasis. For the CN0 assessment criteria, the clinical examination did not address enlarged LNs or swollen LNs that had a soft texture. Ultrasound examination showed no enlarged or swollen LNs that were oval and flat, with clear boundaries between the cortex and medulla, regularly shaped, or had a clear central fat hilum and no obvious malignant signs. We excluded not-PTC, cN1 PTC, reoperation, and patients without LND.

### Study Design

**Figure 1:**
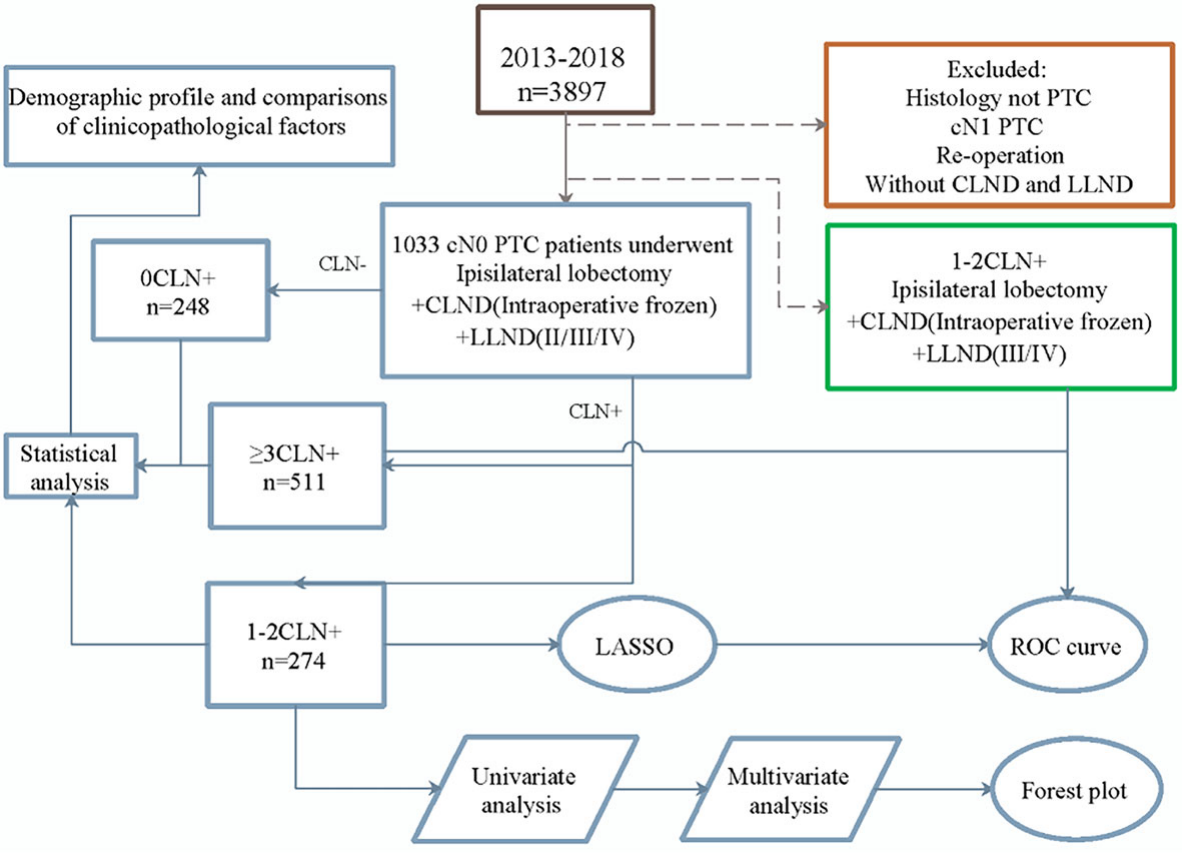
Overall flowchart of the study design.

The research process is shown in **Figure 1**. According to the number of CLNMs, patients were divided into three groups: negative, one or two positive, or over three positive. Univariate analysis was performed to explore the differences in demographic and clinicopathological characteristics among the groups.

Univariate and multivariate analyses were carried out with patients in the 1–2 CLNM group to determine risk factors associated with LLNM. The LLNM risk prediction model was constructed using the least absolute shrinkage and selection operator (LASSO) method. The method shows good stability and effectively avoids overfitting of the model (19). The risk score was estimated by the sum of the value of each factor multiplied by its corresponding coefficient (α). The standard formula is A1 × α1 + A2× α2 +…An × αn. Every single patient was calculated with a risk score and divided into high- or low-risk groups according to the median score. Meanwhile, to evaluate the quality of the model, time-dependent receiver operating characteristic (ROC) analysis was performed to determine the area under the curve (AUC).

Sex (male, female), age of diagnosis, age (≤30 years, >30 years), tumor size (≤10 mm, >10 mm and ≤20 mm, >20 mm), tumor location (upper/middle/lower pole), nodule number, bilaterality, calcification (microcalcification within thyroid nodules), extrathyroidal extension (ETE), Hashimoto’s thyroiditis (HT), thyroid adenoma, follicular variant, multifocality, CLNM number (2/1), the location of CLNM (prelaryngeal, pretracheal, paratracheal LNM and lymph node posterior to right recurrent laryngeal nerve/LN-prRLN metastasis), number of metastatic and harvested CLNs, number of metastatic and harvested LLNs, and risk (high/low) were included in the analysis.

### Statistical Analysis

Continuous variables were analyzed by t test, and categorical variables were analyzed by the chi-squared or Fisher exact test with SPSS version 25.0 (SPSS Inc., Chicago, IL, United States). A *p*-value <0.05 was considered statistically significant. The LASSO method and ROC curve were applied with the glmnet package and timeROC R package (version 0.3), respectively.

## Results

### Clinicopathologic Characteristics of CLM

CLM was correlated with sex, age of diagnosis, tumor size, ETE, bilaterality, LLNM, and number of harvested CLNs and LLNs (*p* < 0.001) (**Table 1**). Specifically, more patients in the CLMs greater than 3 group had a large tumor size, HT, and bilaterality than those in the other two groups (*p* < 0.001). Patents with 1–2 CLMs had greater calcification than the other groups (35.77% *vs*. 10.89%, 35.77% *vs*. 16.83%), with a nonlinear trend. Differences in LLNM incidences were obvious among the 0, 1–2, and ≥3 CLNM groups (16.53% *vs*. 41.61% *vs*. 64.58%, *p* < 0.001). Based on ATA guidelines, a linear increase in medium–high recurrence risk was observed (29.27% *vs*. 37.59% *vs*. 71.82%, respectively; *p* < 0.001). Two patients experienced recurrence due to LNM in the ≥3 CLNM groups.

**Table 1:** Demographic profile and comparisons of clinicopathological factors of the cohort based on the number of CLN-positive cN0 PTC patients.

	0 CLN	1–2 CLNs	≥3 CLNs	p value
	n = 248	n = 274	n = 511	
Female	211 (85.08%)	189 (68.98%)	346 (67.71%)	<0.001
Age of diagnosis	45.55 ± 12.55	42.06 ± 12.60	39.94 ± 11.42	<0.001
Size	13.17 ± 8.91	13.88 ± 10.62	16.25 ± 9.73	<0.001
Hashimoto’s thyroiditis	35 (14.11%)	50 (18.25%)	120 (24.48%)	0.007
Extrathyroidal extension	27 (10.89%)	58 (21.17%)	84 (16.44%)	0.007
Bilaterality	31 (12.50%)	31 (11.31%)	133 (26.03%)	<0.001
Calcification	67 (27.02%)	98 (35.77%)	86 (16.83%)	<0.001
Metastatic number of CLN	0	1.46 ± 0.50	5.92 ± 3.43	<0.001
Harvested number of CLN	10.13 ± 4.33	10.98 ± 6.25	14.28 ± 6.68	<0.001
Metastatic number of LLN	0.25 ± 0.66	1.19 ± 2.20	2.62 ± 3.62	<0.001
Harvested number of LLN	18.67 ± 4.03	18.68 ± 10.03	22.02 ± 13.35	<0.001
Recurrence	0	0	2	
ATA high and median	48 (19.35%)	103 (37.59%)	367 (71.82%)	<0.001
Positive LLNs of patients	41 (16.53%)	114 (41.61%)	330 (64.58%)	<0.001
Female	34 (89.93%)	79 (62.3%)	225 (67.98%)	<0.001
Age of diagnosis	46.29 ± 15.44	41.12 ± 13.39	39.34 ± 11.56	0.003
Size	17.81 ± 13.45	15.96 ± 10.95	17.93 ± 9.89	0.218
Hashimoto’s thyroiditis	7 (17.07%)	15 (18.99%)	89 (26.89%)	0.007
Extrathyroidal extension	8 (19.51%)	28 (24.56%)	58 (17.58%)	0.267
Bilaterality	6 (14.63%)	16 (14.03%)	92 (27.88%)	0.004
Calcification	11 (26.83%)	48 (42.10%)	63 (19.10%)	<0.001
CLNMs	0	1.52 ± 0.50	6.49 ± 3.81	<0.001
CLNDs	9.88 ± 4.48	9.97 ± 6.36	14.16 ± 6.89	0.009
LLNMs	1.51 ± 0.84	2.82 ± 2.63	4.01 ± 3.81	<0.001
LLNDs	23.05 ± 9.94	19.92 ± 10.08	24.02 ± 12.93	<0.001
ATA high and median	12 (29.27%)	60 (52.63%)	288 (87.27%)	<0.001
Recurrence	0	0	2	

CLN, central lymph node; LLN, lateral lymph node; LNM, lymph node metastasis; ATA, American Thyroid Association; CLNM, central lymph node metastasis; LLNM, lateral lymph node metastasis; CLND, central lymph node dissection; LLND, lateral lymph node dissection.

### Clinicopathologic Characteristics of LLNM for Patients With 1–2 CLNMs

**Figure 2:**
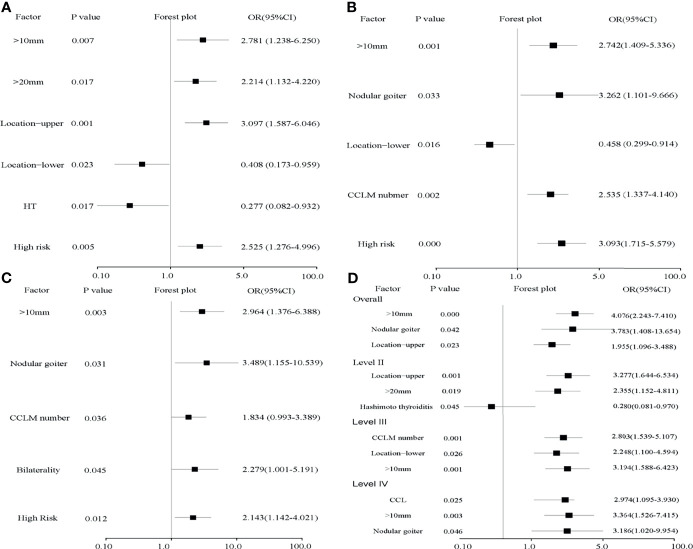
Forest plots of risk factors for levels II–IV **(A–C)** and independent risk factors for LLNM **(D)**. **(A)** Tumor sizes over 20 mm and located in the upper region contribute to level II LLNM and HT reduces its risk. **(B, C)** Tumor size over 10 mm, nodular goiter, and the number of CLNMs were risk factors for level III (*p* = 0.001, OR = 2.742; *p* = 0.033, OR = 3.262; *p* = 0.002, OR = 2.535) and level IV (*p* = 0.003, OR = 2.964; *p* = 0.031, OR = 3.489; *p* = 0.036, OR = 1.834). High risk evaluated by the LASSO model is an independent risk factor for all levels. **(D)** Tumor size over 10 mm, nodular goiter, and upper location were independent risk factors for LLNM. In detail, tumor size over 20 mm and located in the upper region are independent risk factors and HT is an independent protective factor for level II. Nodular goiter is an independent risk factor for level IV.

In this cohort, there was no difference between the LLN (+) and LLN (-) groups in terms of sex, age of diagnosis, nodule number, bilaterality, ETE, thyroid adenoma, follicular variant, multifocality, or location and number of CLNMs (**Table 2** and **Figure S1**). However, patients ≤30 years old had a higher risk of LLNM (*p* = 0.008, OR = 2.303). The number of CLNMs was related to level III (*p* = 0.002, OR = 2.535) and level IV (p = 0.036, OR = 1.834) LLNMs rather than level II (**Table 3** and **Figure 2**). No recurrence was observed in 1–2 CLNM patients who underwent LLND, although one case of recurrence was observed in the LLND (-) group (**Table S2**).

**Table 2:** Univariate analysis of one to two CLNM patients with and without LLNM.

	Total (n = 274)	LLN(+) (n = 114)	LLN(-) (n = 160)	p value	OR	95% CI
Sex(male/female)	85/189	35/79	50/110	0.515	0.975	(0.580–1.639)
Age (mean ± SD, years)	42.06 ± 12.60	41.12 ± 13.39	42.74 ± 11.99	0.298		
(≤55/>55, years)	202/72	81/33	121/39	0.239	1.264	(0.735–2.174)
(>45/45, years)	124/150	52/88	72/66	0.509	1.025	(0.633–1.661)
(30/>30, years)	46/228	27/87	19/141	0.008	2.303	(1.208–4.389)
Tumor size (mm)	13.88 ± 10.62	15.96 ± 10.95	12.40 ± 10.15	0.006		
(>10/10, mm)	180/94	94/20	86/74	<0.001	4.044	(2.278–7.180)
(>20/20, mm)	71/203	41/73	30/130	0.001	2.434	(1.402–4.224)
Location (upper/middle	72/118/78/6	38/47/24/5	34/71/54/1			
/lower/whole)						
(upper/not upper)	72/196	38/71	34/125	0.011	1.968	(1.139–3.399)
Nodule number (1/2/3/>3)	126/71/20/57	55/26/10/23	71/45/10/34	0.695		
Bilaterality (yes/no)	31/243	16/93	15/145	0.157	1.578	(0.746–3.340)
Calcification	98/176	48/66	50/110	0.043	1.600	(0.970–2.638)
Extrathyroidal extension	58/217	28/87	30/130	0.156	1.411	(0.788–2.526)
Hashimoto’s thyroiditis	50224	15/99	35/125	0.045	0.541	(0.280–1.047)
Thyroid adenoma	19/225	10/104	9/151	0.22	1.631	(0.634–4.107)
Nodular goiter	14/260	10/104	4/156	0.021	3.750	(1.146–12.275)
Follicular variant	5/269	3/111	2/158	0.344	2.135	(0.351–12.989)
Multifocality	55/219	24/90	31/129	0.423	1.110	(0.611–2.016)
CLNM number (2/1)	130/144	61/53	69/91	0.058	1.518	(0.936–2.461)
Prelaryngeal LNM	39/235	18/96	21/139	0.326	1.241	(0.628–2.453)
Pretracheal LNM	109/165	44/70	65/95	0.416	0.919	(0.562–1.502)
Paratracheal LNM	111/163	42/72	69/91	0.179	0.769	(0.470–1.259)
LN-prRLN metastasis	10/264	7/107	3/157	0.064	3.424	(0.886–13.536)
Metastatic number of CLN	1.46 ± 0.50	1.52 ± 0.50	1.42 ± 0.50	0.131		
Harvested number of CLN	10.98 ± 6.25	9.97 ± 6.36	11.68 ± 6.10	0.027		
Metastatic number of LLN	1.19 ± 2.20	2.82 ± 2.63	0.03 ± 0.33	<0.001		
Harvested number of LLN	18.68 ± 10.03	19.92 ± 10.08	17.83 ± 9.93	0.091		
LNM number	2.67 ± 2.335	4.36 ± 2.77	1.45 ± 0.57	<0.001		
LND number	29.78 ± 12.433	30.02 ± 12.23	29.61 ± 12.62	0.797		
Risk (high/low)	138/136	79/35	59/101	<0.001	3.864	(2.317–6.444)

CLNM, central lymph node metastasis; LLNM, lateral lymph node metastasis; LNM, lymph node metastasis; LN-prRLN, lymph nodes posterior to the right recurrent laryngeal; LND, lymph node dissection.

**Table 3:** Risk factors for LLNM in the level II, III, and IV groups.

	Total	LNM (+)	LNM (-)	Univariate						Multivariate					
	n = 274	n = 114	n = 160	p		OR 95% (CI)				p	OR 95% (CI)				
Risk High/low	138/136	79/35	59/101	<0.001		3.864		(2.317–6.444)		<0.001	3.864		(2.317–6.444)		
		Level II (+)	Level II (-)												
		n = 45	n = 259												
Tumor size															
>10 mm	180/94	37/8	143/96	0.007		2.781		(1.238–6.250)							
>20 mm	71/203	18/27	53/176	0.017		2.214		(1.132–4.220)		0.019	2.355		(1.152–4.811)		
HT	50/224	3/42	47/182	0.017		0.277		(0.082–0.932)		0.045	0.28		(0.081–0.970)		
Location	6 (whole)														
Upper (yes/no)	72/196	21/23	51/173	0.001		3.097		(1.587–6.046)		0.001	3.277		(1.644–6.534)		
Lower (yes/no)	78/190	7/37	71/153	0.023		0.408		(0.173–0.959)							
Risk															
High/low	138/136	31/14	107/122	0.005		2.525		(1.276–4.996)							
		Level III (+)	Level III (-)												
		n = 68	n = 206												
Tumor size >10 mm	180/94	55/13	125/81	0.001		2.742		(1.409–5.336)		0.001	2.803		(1.539–5.107)		
Nodular goiter	14/260	7/61	7/199	0.033		3.262		(1.101–9.666)							
CLNM(2/1)	130/144	43/25	87/119	0.002		2.535		(1.337–4.140)		0.001	3.194		(1.588–6.423)		
Location Lower (yes/no)	78/190	12/54	66/136	0.016		0.458		(0.299–0.914)		0.026	2.248		(1.100–4.594)		
Risk High/low	138/136	48/20	90/116	<0.001		3.093		(1.715–5.579)							
		Level IV(+)	Level IV (-)												
		n = 52	n = 222												
Tumor size >10 mm	180/94	43/7	137/85	0.003		2.964		(1.376–6.388)		0.003	3.364		(1.526–7.415)		
Nodular goiter	14/260	6/46	8/214	0.031		3.489		(1.155–10.54)		0.046	3.186		(1.020–9.954)		
CLNM(2/1)	130/144	31/21	99/123	0.036		1.834		(0.993–3.389)		0.025	2.974		(1.095–3.930)		
Bilaterality	31/243	10/42	21/201	0.045		2.279		(1.001–5.191)							
Risk High/low	138/136	34/18	104/118	0.012		2.143		(1.142–4.021)							

LLNM, lateral lymph node metastasis; HT, Hashimoto’s thyroiditis; CLNM, central lymph node metastasis.

Tumors were larger in the LLNM group (15.96 ± 10.95 *vs*. 12.40 ± 10.15, *p* = 0.006). Calcification (*p* = 0.043, OR = 1.600) and nodular goiter (*p* = 0.021, OR = 3.750) were positively associated with LLNM. Tumors located in the upper region (*p* = 0.011, OR = 1.968) were also positively related to LLNM, especially for level II (*p* = 0.001, OR = 3.097). Conversely, HT was negatively related to LLNM (*p* = 0.045, OR = 0.541), especially for level II (*p* = 0.017, OR = 0.277) (**Figures 2A, D**). In multivariate analysis, tumor size over 10 mm, tumor located in the upper region, and nodular goiter were independent risk factors for LLNM. In detail, tumor over 20 mm was an independent risk factor for level II (*p* = 0.019, OR = 2.355). Non-microcarcinoma and number of CCLMs were independent risk factors for levels III and IV (**Table 3** and **Figures 2B, C**). Meanwhile, an increased number of CLND was associated with LLNM (*p* = 0.027).

### LASSO Model Construction for Risk Stratification of LLNM

**Figure 3:**
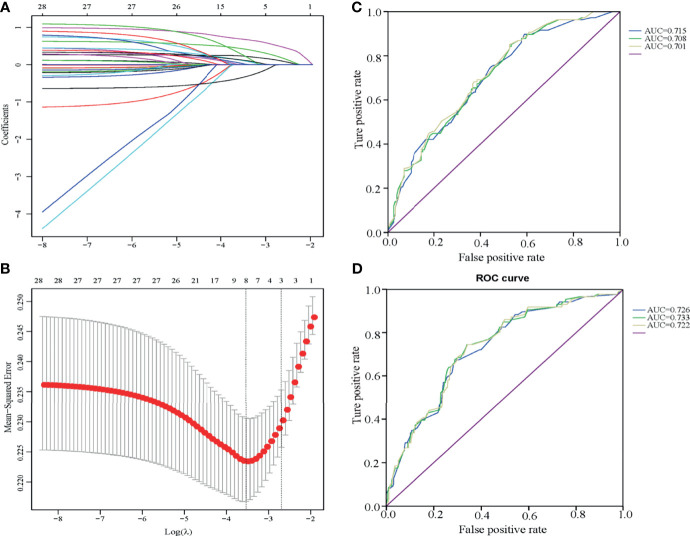
Multivariate risk model constructed by LASSO regression **(A, B)** and ROC curves of the LLNM risk scores for cN0 PTC patients with one to two CLNMs **(C, D)**. **(A, B)** The LASSO model is best constructed with eight factors when Log(λ) equals to -3.5. The eight factors were tumor size 1 (over 10 mm), tumor size 2 (over 20 mm), tumor location, HT, calcification, bilaterality, CLNM number, and nodular goiter. **(C)** The ROC curve of patients who underwent LLND (levels II, III, and IV) presented AUCs of 0.715 (preoperative assessment group with six factors, blue line), 0.708 (intraoperative frozen group with CLNM number enrolled, green line), and 0.701 (postoperative assessment group, yellow line). **(D)** The ROC curve of patients who underwent LLND (levels III and IV) presented AUCs of 0.726 (preoperative assessment group with six factors, blue line), 0.733 (intraoperative frozen group, green line), and 0.722 (postoperative assessment group, yellow line).

All characteristics were analyzed by LASSO (**Figure 3**). Eight LLNM-related factors were identified including tumor size >10 mm, tumor sizes >20 and 10–20 mm, location (upper, middle, and lower), HT, calcification, bilaterality, CLNM number, and nodular goiter (**Table 4**). The coefficients of all risk factors were used to calculate the risk score for each patient as follows: (0.6863518 × A + 0.23244186 ×B +…+H × 0.27637063)/1.55237294. Next, patients whose risk score was lower than 0.8729 were assigned to the low-risk group, and the remaining patients were assigned to the high-risk group. The incidence of LLNM in the high-risk group was 57.25% (79/138), and the risk score was an independent risk factor for LLNM (*p* < 0.001, OR = 3.864). The ROC curve is illustrated in **Figure 3**, and the AUC was 0.715 (0.654–0.776, 95% CI), with a specificity of 0.604 and sensitivity of 0.729 (**Figure 3C**). We excluded CLNM number and nodular goiter for a better preoperative prediction of cN0 PTC patients, and the AUC was 0.701 (0.639–0.764, 95% CI). If intraoperative frozen CLNs were available, the AUC of intraoperative prediction was 0.708 (0.647–0.770, 95% CI). In addition, 479 cN0 PTC patients had only level III and IV LLN and were also enrolled to verify the model (**Figure 3D** and **Table S1**), with an AUC of 0.732 (0.674–0.789, 95% CI). The AUCs of the preoperative prediction and intraoperative prediction models were 0.726 (0.668–0.784, 95% CI) and 0.733 (0.676–0.791, 95% CI), respectively.

**Table 4:** Correlation factors and coefficients of the LASSO model.

	Score	Coefficient
Tumor size1	A1	0.6863518
>10 mm	1	
≤10 mm	0	
Tumor size2	A2	0.23244186
>20 mm	2	
10–20 mm	1	
≤10 mm	0	
Location	A3	0.42974372
Upper	2	
Middle	1	
Lower	0	
Hashimoto’s thyroiditis	A4	-0.34728595
Yes	1	
No	0	
Calcification	A5	0.11499484
Yes	1	
No	0	
Bilaterality	A6	0.03446790
Yes	1	
No	0	
CLNM number	A7	0.12528814
2	2	
1	1	
Nodular goiter	A8	0.27637063
Yes	1	
No	0	
Risk score = (0.6863518 × A + 0.23244186 ×B +…+H × 0.27637063)/1.55237294		

LASSO, least absolute shrinkage and selection operator; CLNM, central lymph node metastasis.

We also verified the model in the ≥3 CLN groups with tumor size >10 mm, tumor sizes >20 and 10–20 mm, location (upper, middle, and lower), HT, calcification, bilaterality, and nodular goiter, and the AUC was 0.716 (0.667–0.765, 95% CI) (**Figure S2**). We ruled out nodular goiter for preoperative prediction because pathological confirmation is needed after surgery. Moreover, the AUC was 0.721 (0.672–0.770, 95% CI). These data confirm that these LASSO models are suitable for patients with CLNM.

## Discussion

PTC has a high morbidity and a low mortality. PTC patients often experience LNM, which is associated with local recurrence (20). Generally, LNM has no significant effect on patient outcome. However, a recent study indicated that it does have a negative impact on survival in high-risk patients (5, 21). LNM is more likely to present a sequential pattern from central to lateral compartments, except in a minority of patients who have skip metastases (22). Currently, CLNM has argues on outcomes. In our study, the increasing number of CLNMs tended to promote regional recurrence and was related to the median (21–36%) and high stratification (68%) of recurrence risk (ATA guidelines for the diagnosis and treatment of thyroid cancer) (12). However, a survival benefit was not observed within the limited time of follow-up. There were also no benefits for cN0 PTC patients who were identified without pathological LNM. Instead, pCLND may increase complications. LLNM has been included as a risk factor for structural recurrence in ATA (12)and has even been shown to influence outcome in some reports (23). As an essential risk factor for LLNM, the number of CCLMs is increasingly being included in risk stratification, with typical cutoff values of 2–3 and 5 (18, 24, 25). We identified number of CLNMs as an independent risk factor for LLNM when patients were stratified into 0, 1–2, and ≥3 CLNM groups. Among them, the skip metastasis risk is 16.53%. In the ≥3 CLNM group, the incidence of LLNM was 64.58% and 71.82% of these patients had a medium–high ATA risk, which should alert clinicians to the possibility of an over 20% local recurrence risk. In the 1–2 CLNM group, the incidences of LLNM and the medium–high recurrence risk were 41.61% and 37.59%, respectively, which were neither high nor low compared to the other groups. Further risk stratification in this group is warranted.

Patients with stage cN0 PTC have a high incidence of occult LNM (26). Experts disagree on the use of prophylactic LND, especially between Western and East Asia. The latter suggests routine pCLND, and the former shows individual variance in pCLND (27). On the one hand, it has been reported that pCLND does not improve outcomes but rather can cause more complications, such as parathyroid and laryngeal nerve injury (28). On the other hand, pCLND has been confirmed to improve the disease-free survival of patients with intermediate and high-risk ATA risk stratification (29).Moreover, pCLND can help with tumor staging, predict LLNM, guide adjuvant radioiodine, reduce postoperative serum thyroglobulin, and decrease the complications of reoperation (30, 31). Indeed, the fifth National Audit Report reports that 37% of patients undergo CLND (32). We performed routine pCLND, and intraoperative frozen pathology results were available. The increasing number of CLNMs was correlated with male sex, younger age, greater tumor size, extrathyroidal extension, bilaterality, calcification, LLNM, and a median-high ATA risk, consistent with previous research (29, 33–36). According to our data, pCLNM can help in effectively screening occult CLNM with an incidence of 75.99% and assess the risk of occult LLNM.

In general, experts do not recommend pLLND for cN0 PTC patients. Some of them perform LLND for high-risk LLNM patients based on clinical features and experience. The number of LNMs helps improve tumor staging and postoperative treatment. There have been numerous risk models to assess LLNM with different methods (25, 37). However, consistency in terms of the LND extent was lacking among the enrolled patients and the results were not precise enough to confirm negative LLNs by imaging and palpation. The traditional extent of LLND is levels II–V, but the debate over level IIb and V dissection remains ongoing due to spinal accessory nerve injuries. Some studies argue that there is no difference in recurrence rates between selective (including II, III, and IV) and traditional neck dissection (38). The incidence of metastasis with level V dissection is low, but the rate of shoulder dysfunction is high (39). Meanwhile, level II is associated with skip metastasis (16), and the incidence of shoulder syndrome is low. Therefore, we adopted levels II–IV as the extent of LND and the incidence of occult LLNM was 46.95%.

In the 1–2 CLNM group, we found that age ≤30 years, tumor size over 10 mm, tumors in the upper region, calcifications, and nodule goiters contributed to LLNM. Hashimoto’s thyroiditis reduced the risk of LLNM. Liu et al. indicated that patients (age ≤30 years old) are susceptible to local recurrence and that patients (<45 years old) with lateral neck LNM are understaged by the 7th edition of the AJCC staging system (40). Our data also showed that 50% (11/22) of patients (age ≤30 years old) with LLNM belong to the medium–high ATA risk group. For this population, genetic testing is a good method for individualized evaluation, such as the RET gene (41). Tumors over 10 mm and those located in the upper region are acknowledged as independent risk factors for LLNM (42). We also confirmed these findings and further reveal that tumor size greater than 20 mm and a tumor located in the upper region are independent risk factors for level II disease. Tumors located in the lower region were an independent risk factor for level III tumors. It has been reported that nodular goiter is an independent risk factor for LLNM (25), which supports our findings. Nonetheless, more data is needed to prove this hypothesis, and the mechanism remains unclear. HT is a controversial topic as some argue that HT has a negative effect on LNM (43), whereas others assert that it has no connection with LNM (44). Some have even argued that its adverse effects on CLNM and antibody status were risk factors of CLNM with a cutoff of 3 (45). In our research, HT was related to level II LLNM (*p* = 0.045, OR = 0.280) rather than level III or IV. Moreover, HT showed no relationship with LLNM in patients with 1–2 CLNMs who underwent level III and IV LLND (*p* = 0.399). We found that the morbidity of CLNM in patients with HT was higher than that in patients without HT (170/205 *vs.* 615/828), although HT showed no connection with the number of positive CLNs in subsequent analyses. We also found that HT was negatively related to ETE (*p* = 0.040, OR = 0.444). One recurrent male who did not undergo LLN dissection was found to have level III–IV LN metastasis and BRAF, TERT, and PIK3CA mutations. No cases of relapses were observed in the LLND (+) group (**Table S2**). Survival benefits were not observed for LLND, and recurrence was not common in 1–2 CLNM cN0 PTC patients without LLND. The results show that routine dissection of the lateral lymph nodes is unnecessary, but effort to screen people at high-risk people for occult LLNM should be considered.

We constructed the LASSO model and divided people into low- (25.74%) and high-risk (57.25%) LLNM groups. In the high-risk group (risk score over 0.8729), 78.38% of patients had a median and high ATA risk. LASSO risk stratification is an independent risk factor for LLNM. It aids in the preoperative assessment of LLNM risk for cN1a PTC patients using six factors. Intraoperative frozen pathology results also help intraoperative decisions on pLLND combined with clinical experience to guides postoperative therapy, such as radioactive iodine treatment and TSH inhibition. For high-risk patients, we selectively suggest a polygenetic test to further screen for higher aggressiveness of PTC and identify potential therapeutic targets for subsequent recurrence (46). The results show that RET, TERT, PIK3CA, and fusion mutations are common (**Table S3**) and longer follow-up periods are needed to determine whether LLND reduces regional recurrence in the high-risk population.

There are several limitations to this study that should be mentioned. Although the rate of occult level II was 16.42%, selection bias regarding the extent of LLND between the III–IV and II–IV groups might exist among surgeons, leading to the relatively high rates of occult LLNM in the latter group. We herein present postoperative TG monitoring, tumor persistence, local recurrence, distant metastasis, and survival data to determine whether LLND is meaningful or harmful for high-risk patients, but long-term follow-up data are also needed.

In summary, for patients with 1–2 CLNMs, young age, calcification, nodular goiter, tumor >10, and tumor in the upper region are risk factors for LLNM. High-risk patients in the LASSO model had a 57.25% chance of occult LLNM and a 78.38% chance of having moderate–high ATA risk. These findings may serve as a reference for selective pLLND, comprehensive therapy, and follow-up.

## Data Availability Statement

The original contributions presented in the study are included in the article/**Supplementary Material**. Further inquiries can be directed to the corresponding authors.

## Ethics Statement

This study was approved by the Ethics Committee of the First Affiliated Hospital of Chongqing Medical University, and each participating patient provided written informed consent.

## Author Contributions

YW and XLS conceptualized and designed the study. YW wrote the manuscript and approved the final manuscript. JT provided the study material. CD, HW, XJS, and PY collected the data and specimen. YW and JT analyzed and interpreted the data. All authors contributed to the article and approved the submitted version.

## Conflict of Interest

The authors declare that the research was conducted in the absence of any commercial or financial relationships that could be construed as a potential conflict of interest.

## Publisher’s Note

All claims expressed in this article are solely those of the authors and do not necessarily represent those of their affiliated organizations, or those of the publisher, the editors and the reviewers. Any product that may be evaluated in this article, or claim that may be made by its manufacturer, is not guaranteed or endorsed by the publisher.

## Supplementary Material

Supplementary Figure 1 The forest plot of risk factors for LLNM. Sex, age, bilaterality, ETE, thyroid adenoma, follicular variant, multifocality, CLNM number and location were not related to LLNM for cN0 PTC patients with 1-2CLNMs. Tumor size over 10 mm (*p*=0.000, OR=4.004), upper tumor location (*p*=0.011, OR=1.968), calcification (*p*=0.043, OR=1.600), nodular goiter (*p*=0.021, OR=3.750) and high risk (*p*=0.000, OR=3.864) were risk factors of LLNM. HT (*p*=0.045, OR=0.541) was a protective factor against LLNM.

Supplementary Figure 2 ROC curves of LLNM risk scores for cN0 PTC with ≥3 CLNMs. The ROC curve of patients who underwent LLND (levels II, III and IV) presented AUCs of 0.721 (preoperative assessment group with six factors, blue line) and 0.716 (postoperative assessment group with seven factors, CLNM number excluded, green line).

## Abbreviations

TC, thyroid cancer; cN0, clinically lymph node-negative; PTC, papillary thyroid carcinoma; LNM, lymph node metastasis; LND, lymph node dissection; CLNM, central lymph node metastasis; LLNM, lateral lymph node metastasis; NCCN, National Comprehensive Cancer Network; ATA, American Thyroid Association; pCLND, prophylactic central lymph node dissection; pLLND, prophylactic lateral lymph node dissection; DTCC, Database Technology Conference China; FNAB, fine-needle aspiration biopsy; LASSO, least absolute shrinkage and selection operator; ROC, time-dependent receiver operating characteristic; AUC, the area under the ROC curve; ETE, extrathyroidal extension; HT, Hashimoto’s thyroiditis.
