# Supplementary figures and images for: Risk Factors and a Prediction Model of Lateral Lymph Node Metastasis in CN0 Papillary Thyroid Carcinoma Patients With 1–2 Central Lymph Node Metastases
Source: Front Endocrinol (Lausanne). 2021 Oct 15;12:716728. doi: 10.3389/fendo.2021.716728 (PMC8555630; doi:10.3389/fendo.2021.716728)

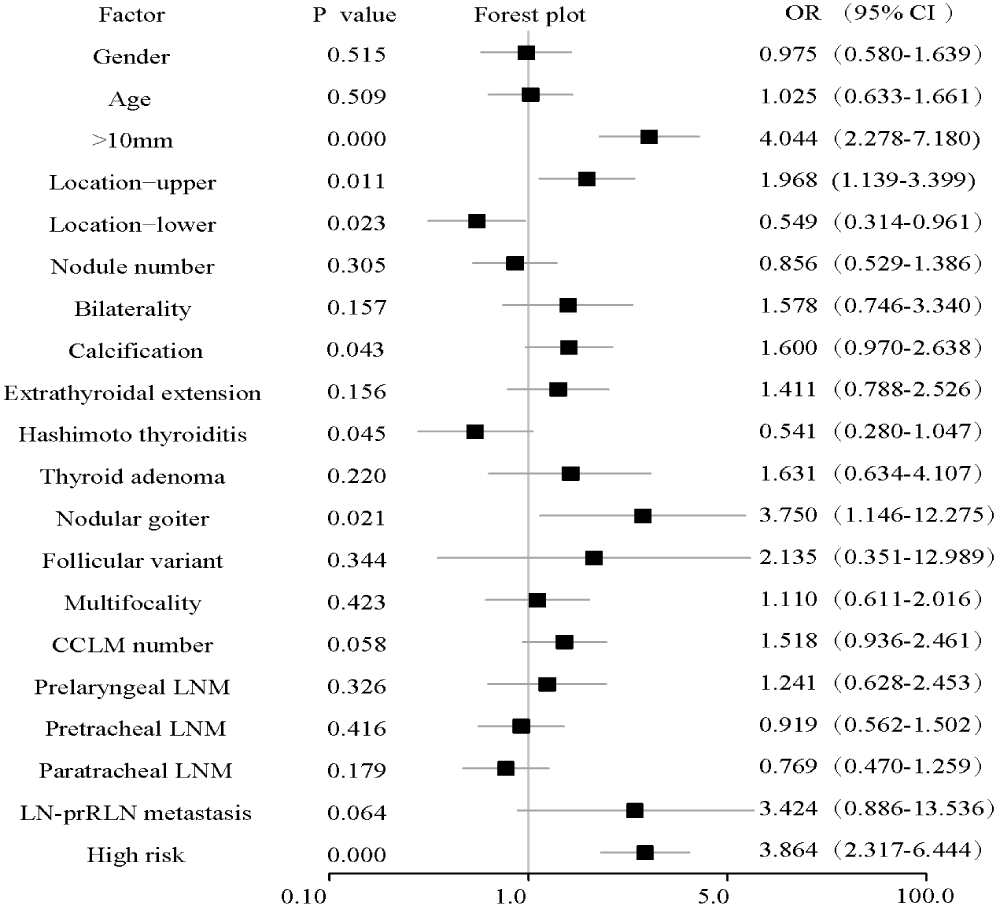

Supplement: Supplementary Figure 1 — The forest plot of risk factors for LLNM. Sex, age, bilaterality, ETE, thyroid adenoma, follicular variant, multifocality, CLNM number and location were not related to LLNM for cN0 PTC patients with 1-2CLNMs. Tumor size over 10 mm (p=0.000, OR=4.004), upper tumor location (p=0.011, OR=1.968), calcification (p=0.043, OR=1.600), nodular goiter (p=0.021, OR=3.750) and high risk (p=0.000, OR=3.864) were risk factors of LLNM. HT (p=0.045, OR=0.541) was a protective factor against LLNM. [file Image_1.tif]

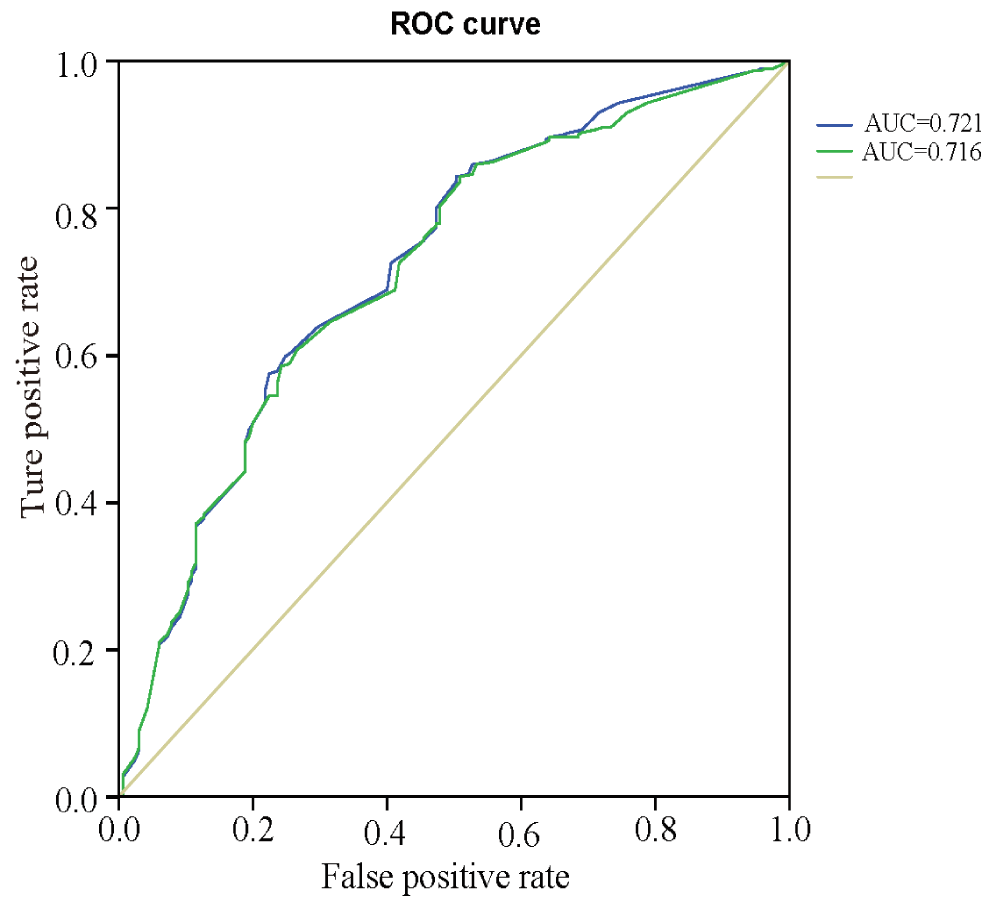

Supplement: Supplementary Figure 2 — ROC curves of LLNM risk scores for cN0 PTC with ≥3 CLNMs. The ROC curve of patients who underwent LLND (levels II, III and IV) presented AUCs of 0.721 (preoperative assessment group with six factors, blue line) and 0.716 (postoperative assessment group with seven factors, CLNM number excluded, green line). [file Image_2.tif]
