# Supplementary material for: Risk Factors and a Prediction Model of Lateral Lymph Node Metastasis in CN0 Papillary Thyroid Carcinoma Patients With 1–2 Central Lymph Node Metastases
Source: Front Endocrinol (Lausanne). 2021 Oct 15;12:716728. doi: 10.3389/fendo.2021.716728 (PMC8555630; doi:10.3389/fendo.2021.716728)
Supplement: Supplementary file 3 [file Table_1.docx]

Table S1 Demographic profile and comparisons of clinicopathological factors of 1–2 CLNM patients between the level II-IV and III-IV groups.

|  |  | II-IV |  | III-IV |
| --- | --- | --- | --- | --- |
|  |  | n=274 |  | n=479 |
| Female |  | 189(68.98%) |  | 349(72.86%) |
| Age of diagnosis |  | 42.06±12.60 | | 42.09±12.06 |
| Size |  | 13.88±10.62 |  | 12.76±8.47 |
| Hashimoto's thyroiditis |  | 50(18.25%) |  | 87(18.16%) |
| Multifocality |  | 55(20.07%) |  | 89(18.58%) |
| Bilaterality |  | 31(11.31%) |  | 73(15.24%) |
| Extrathyroidal extension |  | 58(21.17%) |  | 92(19.21%) |
| Upper |  | 78(28.47%) |  | 102(21.29%) |
| Calcification |  | 98(35.77%) |  | 95(19.83%) |
| Metastatic number of CLN |  | 1.46±0.50 |  | 1.37±0.48 |
| Metastatic number of LLN |  | 1.19±2.20 |  | 0.55±1.77 |
| ATA High&median |  | 103(37.59%) |  | 111(23.17%) |
| Positive LLNs of patients |  | 114(41.61%) |  | 92(19.21%) |
