# Supplementary material for: Risk Factors and a Prediction Model of Lateral Lymph Node Metastasis in CN0 Papillary Thyroid Carcinoma Patients With 1–2 Central Lymph Node Metastases
Source: Front Endocrinol (Lausanne). 2021 Oct 15;12:716728. doi: 10.3389/fendo.2021.716728 (PMC8555630; doi:10.3389/fendo.2021.716728)
Supplement: Supplementary file 4 [file Table_2.docx]

Table S2 Demographic profile and comparisons of clinicopathological factors of the cohort based on LLND for 1–2 CLNM patients.

|  |  | LLND(+) |  | LLND(-) |
| --- | --- | --- | --- | --- |
|  |  | n=274 |  | n=273 |
| Male |  | 85(31.02%) |  | 67(24.54%) |
| Age of diagnosis |  | 42.06±12.60 | | 41.40±11.49 |
| Size |  | 13.88±10.62 |  | 10.40±6.11 |
| Hashimoto's thyroiditis |  | 50(18.25%) |  | 50(18.31%) |
| Location (upper) |  | 72(26.28%) |  | 46(16.85%) |
| Extrathyroidal extension |  | 58(21.17%) |  | 26(9.52%) |
| Bilaterality |  | 31(11.31%) |  | 41(15.01%) |
| Calcification |  | 98(35.77%) |  | 86(31.50%) |
| Multifocality |  | 55(20.07%) |  | 52(19.05%) |
| Metastatic number of CLN |  | 1.46±0.50 |  | 1.32±0.47 |
| Harvested number of CLN |  | 10.98±6.25 |  | 7.36±5.28 |
| Metastatic number of LLN |  | 1.19±2.20 |  | —— |
| Harvested number of LLN |  | 18.68±10.03 |  | —— |
| Recurrence |  | 0 |  | 1 |
