# Supplementary material for: Risk Factors and a Prediction Model of Lateral Lymph Node Metastasis in CN0 Papillary Thyroid Carcinoma Patients With 1–2 Central Lymph Node Metastases
Source: Front Endocrinol (Lausanne). 2021 Oct 15;12:716728. doi: 10.3389/fendo.2021.716728 (PMC8555630; doi:10.3389/fendo.2021.716728)
Supplement: Supplementary file 5 [file Table_3.docx]

Table S3 The polygenetic mutations and clinicopathologic characteristics of high risk cN0 PTC.

|  |  | 1–2CLNMs(+) |  | ≥3CLNMs(+) |
| --- | --- | --- | --- | --- |
|  |  | n=8 |  | n=23 |
| Male |  | 3(37.50%) |  | 9(39.13%) |
| Age of diagnosis |  | 38.78±11.71 | | 34.78±13.56 |
| Size |  | 17.85±22.43 |  | 19.61±8.81 |
| Hashimoto's thyroiditis |  | 0 (0.00%) |  | 7(30.43%) |
| Location (upper&middle) |  | 8 (100.00%) |  | 13 (56.52%) |
| Extrathyroidal extension |  | 5(62.50%) |  | 15(65.22%) |
| Bilaterality |  | 1(12.50%) |  | 12(52.17%) |
| Multifocality |  | 3(37.50%) |  | 6(26.09%) |
| LLNM |  | 7(87.50%) |  | 20(86.96%) |
| Metastatic number of CLN |  | 1.375±1.04 |  | 7.78±2.64 |
| Metastatic number of LLN |  | 4.00±5.85 |  | 1.32±0.47 |
| Metastatic number of LN |  | 5.38±5.75 |  | 5.43±4.69 |
| Gene mutation |  | 7 |  | 23 |
| BRAF |  | 6 |  | 15 |
| Except for BRAF |  | 2 |  | 15 |
| Fusion mutation |  | 1 |  | 4 |
| RET |  | 0 |  | 6 |
| PIK3CA |  | 0 |  | 2 |
| TRET |  | 0 |  | 2 |
| RAC1 |  | 1 |  | 0 |
| ATM |  | 1 |  | 0 |
| NTRK1 |  | 0 |  | 1 |
| ATM |  | 0 |  | 1 |
| CHEK2 |  | 0 |  | 2 |
| PPARG |  | 0 |  | 1 |
| GNAS |  | 0 |  | 1 |
| AXIN1 |  | 0 |  | 1 |
| Recurrence |  | 0 |  | 1 |
